# Supplementary figures and images for: Effects of Stellera chamaejasme removal on the nutrient stoichiometry of S. chamaejasme-dominated grasslands in the Qinghai–Tibetan plateau
Source: PeerJ. 2020 Jun 23;8:e9239. doi: 10.7717/peerj.9239 (PMC7319027; doi:10.7717/peerj.9239)

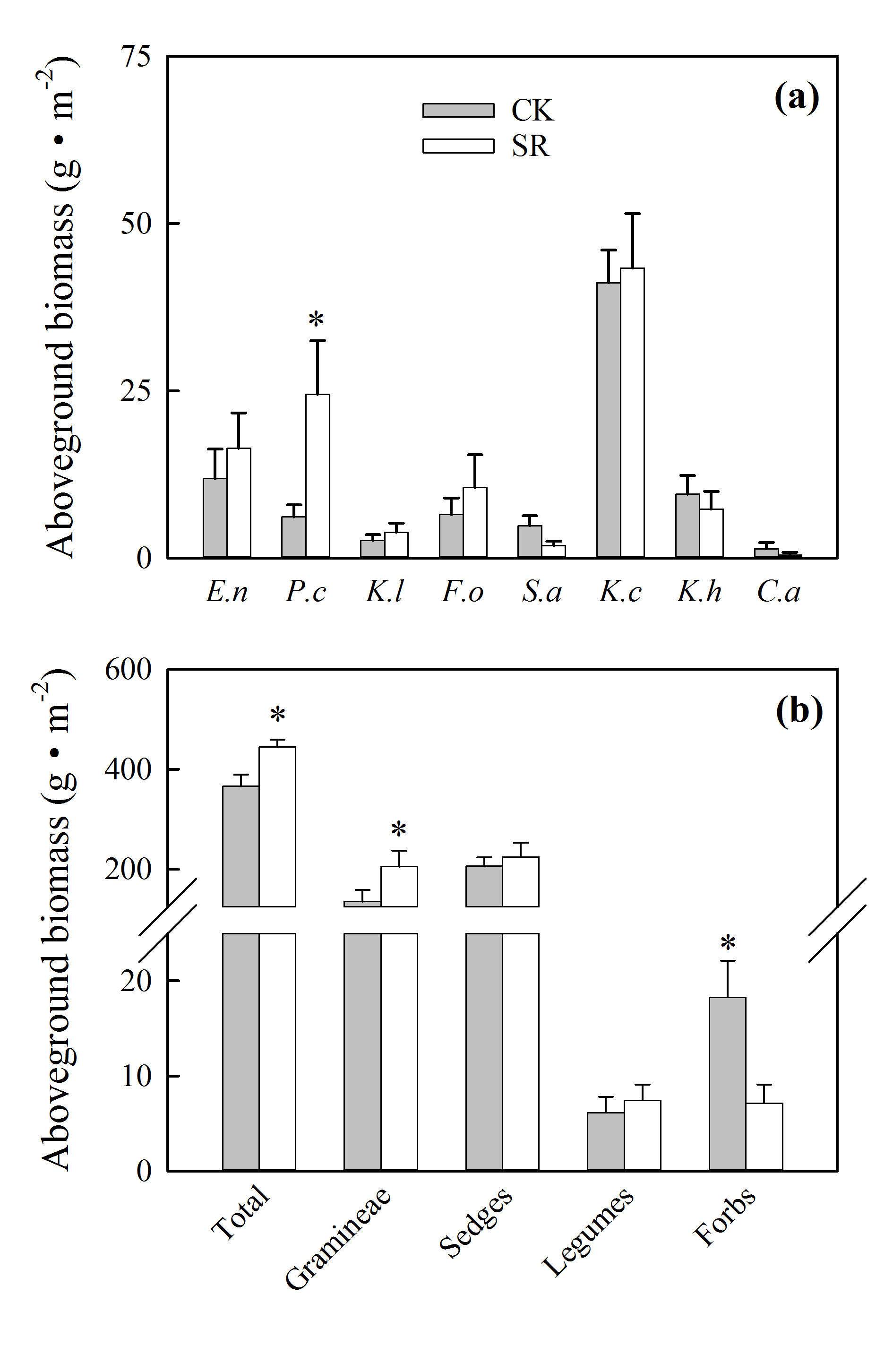

Supplement: Supplemental Information 1 — E.n: Elymus nutans; P.c: Poa crymophila; K.l: Koeleria litvinowii; F.o: Festuca ovina; S.a: Stipa aliena; K.c: Kobresia capillifolia; K.h: Kobresia humilis; C.a: Carex atrofusc.Asterisk (*) denotes a significant difference (P < 0.05). [file peerj-08-9239-s001.png]

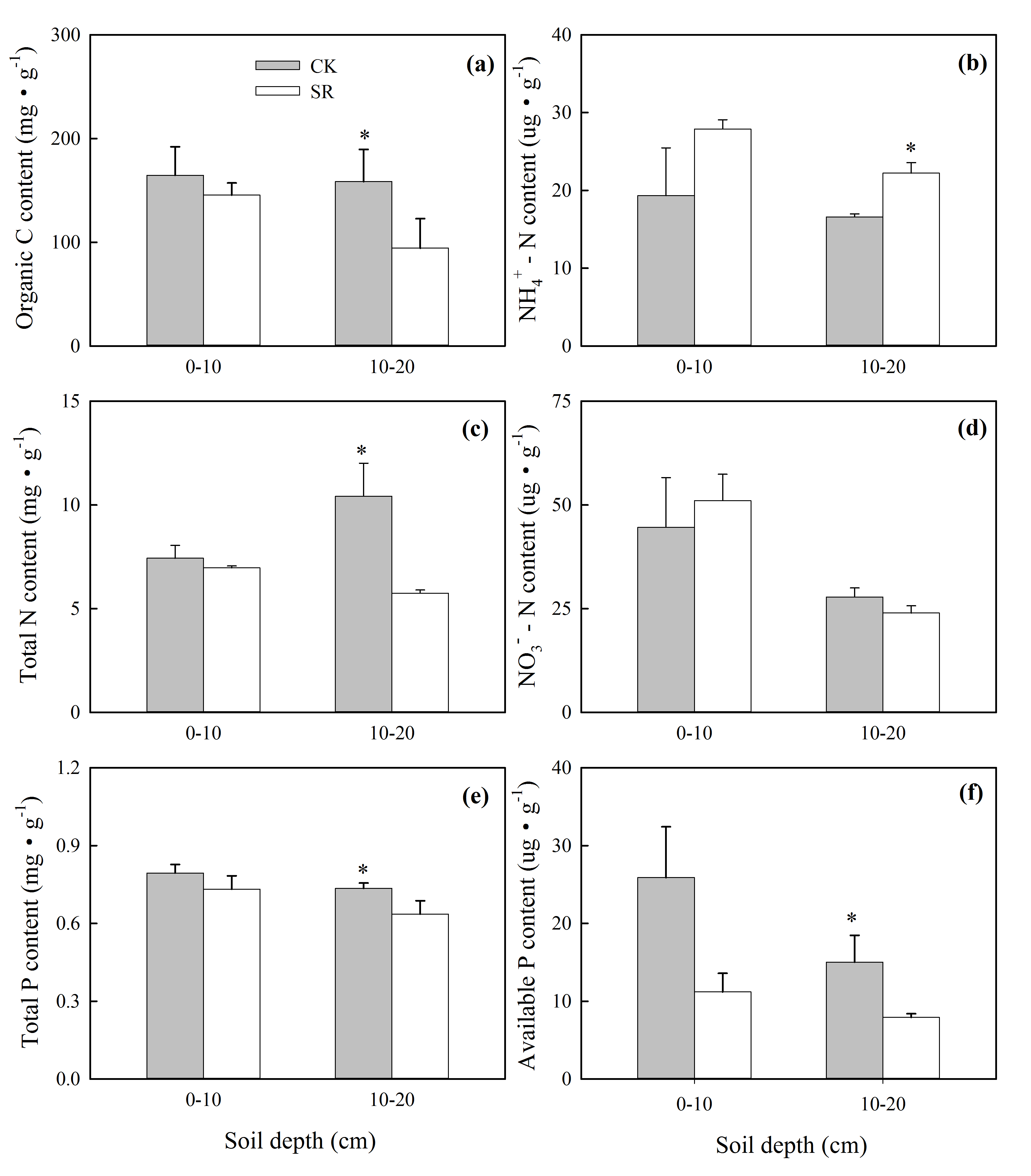

Supplement: Supplemental Information 2 — Asterisk (*) denotes a significant difference (P < 0.05). [file peerj-08-9239-s002.png]
