# Supplementary material for: Effects of Stellera chamaejasme removal on the nutrient stoichiometry of S. chamaejasme-dominated grasslands in the Qinghai–Tibetan plateau
Source: PeerJ. 2020 Jun 23;8:e9239. doi: 10.7717/peerj.9239 (PMC7319027; doi:10.7717/peerj.9239)
Supplement: Supplemental Information 4 — Value shows mean ± SE. Different lowercase letters denote significant differences (P < 0.05) between CK and SR treatments. [file peerj-08-9239-s004.docx]

|  | **Importance value** | | | |
| --- | --- | --- | --- | --- |
| **Species** |  | **CK** |  | **SR** |
| *Elymus nutans* |  | 0.075±0.011 **b** |  | 0.141±0.029 **a** |
| *Poa crymophila* |  | 0.086±0.009 **b** |  | 0.164±0.040 **a** |
| *Koeleria litvinowii* |  | 0.065±0.005 |  | 0.074±0.013 |
| *Festuca ovina* |  | 0.061±0.014 |  | 0.072±0.023 |
| *Stipa aliena* |  | 0.065±0.009 |  | 0.038±0.010 |
| *Kobresia capillifolia* |  | 0.220±0.024 |  | 0.243±0.041 |
| *Kobresia humilis* |  | 0.109±0.017 |  | 0.065±0.022 |
| *Carex atrofusca* |  | 0.011±0.009 |  | 0.011±0.006 |
