# Supplementary material for: Effects of Stellera chamaejasme removal on the nutrient stoichiometry of S. chamaejasme-dominated grasslands in the Qinghai–Tibetan plateau
Source: PeerJ. 2020 Jun 23;8:e9239. doi: 10.7717/peerj.9239 (PMC7319027; doi:10.7717/peerj.9239)
Supplement: Supplemental Information 5 — Different lowercase letters denote significant differences (P < 0.05) between CK and SR treatments. [file peerj-08-9239-s005.docx]

| Treatments | Species richness |  | Shannon-Wiener index |  | Simpson index |  | Pielou index |
| --- | --- | --- | --- | --- | --- | --- | --- |
| CK | 15.17 ± 0.806 **a** |  | 2.27 ± 0.056 **a** |  | 0.86 ± 0.009 **a** |  | 0.84 ± 0.009 |
| SR | 12.42 ± 0.690 **b** |  | 2.00 ± 0.059 **b** |  | 0.81 ± 0.012 **b** |  | 0.80 ± 0.014 |
